# Factors influencing the degree of physician-pharmacists collaboration within governmental hospitals of Jigjiga Town, Somali National Regional State, Ethiopia, 2020

**DOI:** 10.1186/s12913-021-07301-7

**Published:** 2021-11-24

**Authors:** Workineh Diriba Gemmechu, Endalkachew Mekonnen Eticha

**Affiliations:** College of Medicine and Health Science, School of Medicine, Jigjiga University, Jigjiga, Ethiopia

**Keywords:** Physician, Pharmacist, Factors, Collaboration

## Abstract

**Background:**

Collaboration is the way to deliver the desired health outcome for the patients or service users in the healthcare. Inter-professional collaboration can improve medication safety, patient outcome and minimize healthcare costs. This study aimed to explore the degree of collaboration and factors influencing collaboration between physicians and pharmacists within the public hospitals of Jigjiga town, Somali National Regional State, Ethiopia, 2020.

**Methods:**

A cross-sectional study qualitative was conducted among 149 participants in the two governmental hospitals of the Jigjiga town with a response rate of 79.87%. The collaborative working relationship model and the physician-pharmacist collaborative instrument with three main exchange domains (trustworthiness, role specification, and relationship initiation) and collaborative care items were used. An independent sample t-test was used to compute the differences of the mean scores of physician-pharmacist collaborative instrument domains and collaborative care. Separate multiple regression was employed to assess factors influencing collaborative care for pharmacists and physicians.

**Results:**

This study showed that pharmacists reported higher mean of collaborative care (10.66 ± 4.75) than physicians (9.17 ± 3.92). The multiple regression indicated that area of practice influence both professionals’ collaborative practice. A significant association between collaborative care and the two PPCI domains (trustworthiness and relationship initiation for the physicians; role specification and relationship initiation for pharmacists) was established.

**Conclusions:**

The study showed that the collaboration between the physicians and pharmacists was sub-optimal and the exchange variables had a significant influence on their collaboration.

**Recommendation:**

Physicians and pharmacists need to exert more efforts to enhance this collaboration. Further qualitative study might be needed to search for factors affecting, barriers and how to develop collaborative practice.

**Supplementary Information:**

The online version contains supplementary material available at 10.1186/s12913-021-07301-7.

## Background

Collaboration is the way to deliver the desired health outcome for the patients or service users in the healthcare system [1, 2]. According to World Health Organization (WHO): “collaborative practice happens when multiple health workers from different professional backgrounds work together with patients, families, careers and communities to deliver the highest quality of care across settings” [3, 4]. It was felt that the interaction between health care professionals in the past has been limited. The collaboration between pharmacists and physicians is often sub-optimal and less than satisfactory [5, 6]. Sub-optimal relationship between the two aforementioned professionals carriers potential adverse effects on the patients and the healthcare system [7, 8].

Physicians and pharmacists’ collaboration has been shown to improve the outcomes of medication therapy [1, 9]. The inclusion of pharmacists in the physician-pharmacist collaborative care model was associated with significant improvements in patients’ medication-related clinical health outcomes and a reduction in hospitalizations [10, 11]. A study in England revealed that clinical pharmacist-physician collaboration saved critical care patients from more than 500 instances of potential harm [12]. Similar findings were reported among patients with hypertension [13–15] diabetes [16], heart failure [17] and dyslipidemia [18].

As a part of a paradigm shift, Ethiopia has changed to the patient-oriented pharmacy curriculum [19] and has been shown to improve patients outcomes [20–23]. These patient-oriented clinical pharmacy services depend on the concept of pharmaceutical care to improve medication therapy outcomes in collaboration with the physicians. Physicians were the important health professionals for achieving the objectives of clinical pharmacy services [24]. This study was aimed to assess the degree of physician-pharmacists collaboration and factors influencing their collaboration in the two public hospitals’ of Jigjiga town, Somali National Regional State (SNRS), Ethiopia.

## Methods

### Study design, setting and participants

This hospital-based cross-sectional quantitative study design was conducted in two governmental hospitals of Jigjiga town from October to November 2020. According to 2015 central statistical Agency, the population of the town has an estimated total population of 304, 000 of whom 151,422 men and 153, 578 were women. The current population of this city is 304,000. In the Jigjiga town, there are two governmental Hospitals, Karamara Hospital (KH) and Sheik Hassan Yebare Referral Hospital (SHYRH) and three health care centers. The hospitals provide services for greater than one million populations annually.

### Study population

All pharmacists and physicians in both settings were enrolled into the study after the written consent was obtained. Medical Intern students were also included in the study. The list of the participants was recruited from KH and SHYRH Medical Director Database for the employees. Guests and temporary employees of the two settings were excluded.

### Data Collection technique and instruments

A self-administered questionnaire was distributed to pharmacists and physicians after written informed consent was received. The survey had two forms: one for physicians and another for pharmacists. No compensation was offered. Two clinical pharmacists distributed, followed-up and collected the questionnaires. The hospital pharmacy department head of both settings helped in data collection as well by creating a favorable relationship with the study participants to fill and return the questionnaires. The confidentiality of the study participants was maintained by assigning unique identifiers during data collection and analysis.

A conceptual model developed by McDonough and Doucette [25] was used to measure inter-professional collaboration and factors influencing physicians’ and pharmacists’ collaboration. The model was supported by several studies [26–29]. Hence, the collaborative working relationship (CWR) was adopted in this study. The model has three main groups of independent variables that can affect physician-pharmacist collaboration. These include, (1) individual characteristics such as demographics and professional experience of both pharmacist and physician; (2) structure of the health care settings such as pharmacy, hospital and clinic; (3) exchange characteristics among professionals, including trustworthiness, role specification and relationship initiation [26]. These factors could act as barriers or promoters to the collaboration.

The pharmacist-physician collaboration instrument (PPCI) had 14 items that provides a summary score from 14 to 92, with the higher score indicates a greater extent of collaboration. The PPCI was used and calculated for each respondent. The PPCI measures three domains of a collaborative relationship: (1) trustworthiness, a practitioner’s ability to trust another practitioner’s word and expertise; (2) role specification, interactions between pharmacists and physicians in which they reach an agreement on roles and responsibilities for each other in caring for mutual patients; 3) relationship initiation, the actions of one party to determine the needs of another party thereby facilitating relationship development.

A validated 29 14 items of PPCI domains were used to measure the degree of pharmacists-physicians collaboration and factors influencing collaboration in the healthcare team to achieve positive patient outcomes. Items for the two PPCI domains (trustworthiness, role specification) and collaborative care were scored on a 7 point Likert scale ranging from 1 (very strongly disagree) to 7 (very strongly agree). But, items for the relationship initiation were scored on a five-point likert scale (strongly agree to strongly disagree). The score range of the three exchange domain includes 6-42 in the trustworthy domain (the first six questions), 5-35 in the role specification domain (the second five questions), and 3 - 15 in the relationship initiation domain (the last three questions) [29, 30] as provided in the supplementary file 1. The survey also included a five items measure of collaborative care with the score ranges of 5-35. Collaborative care was related to physician-pharmacist collaboration for their common patients.

In addition, to the PPCI, the questionnaire included information regarding pharmacists’ and physicians’ ages, gender, years of experience, practice area and educational level which could potentially influence collaboration.

### Data analysis and interpretation

The collected data were screened, sorted, coded, entered and analyzed by use of SPSS version 25 and presented in the form of frequency tables. Descriptive statistics were used to summarize the frequency and percentages of participants’ demographics. Cronbach’s alpha was calculated for each PPCI domain and collaborative care to determine reliability. An independent sample t-test was used to compute the mean scores of PPCI domains and collaborative care. Separate multiple regression (Generalized linear models) analysis was used to assess factors influencing pharmacist and physician collaboration.

## Results

### Socio-demographic characteristics of the study participants

A total of 149 questionnaires were distributed to the physicians and pharmacists working in both settings (KH and SHYRH). Fifty-two physicians (62.7%) and sixty-seven pharmacists filled and returned the survey with a response rate of 79.9%. Most of the physicians (42, 80.8%) were in the age group of 26-35 years and had ≤ 5 years of experience. Sixteen physicians (30.8%) were practicing in the internal medicine ward. The socio-demographic characteristic of physicians was summarized in Table 1 below.

**Table 1:** Socio-demographic characteristics of the physicians working in the two governmental hospitals of Jigjiga town, SNRS, Ethiopia, November 2020

Variables	Variables Category	Frequency, N (percentage, %)
Age in years	18-25	7 (13.5)
	26-35	42 (80.8)
	36-45	2 (3.8)
	≥46	1 (1.9)
Sex	Male	48 (92.3)
	Female	4 (7.7)
Years of service experience	≤ 5	42 (80.8)
	>5	10 (19.2)
Educational level	GP	42 (80.8)
	Medical Specialist	10 (19.2)
Practice area	Internal medicine ward	16 (30.8)
	Pediatric ward	9 (17.3)
	Surgical ward	8 (15.4)
	OPD ward	9 (17.3)
	Emergency Ward	2 (3.8)
	Gyn and Obs ward.	8 (15.4)

Key: GP, General practitioner; Abbreviation; Gyn, gynecology; Obs, obstetrics;

A large proportion of the pharmacists (53, 79.1%) had ≤ 5 years of practice as shown in Table 2. Nine (13.4%) pharmacists routinely provide clinical pharmacy services, while large percentages of the pharmacists (58, 86.6%) were dispensers. The majority of pharmacists (74.6%) and physicians (92.3%) were males.

**Table 2:** Socio-demographic characteristics of pharmacists working in the governmental hospitals of Jigjiga town, SNRS, Ethiopia, November 2020

Variables	Variable category	N (%)
Age	18-25	32 (47.8)
	26-35	22 (32.8)
	36-45	4 (6)
	≥46	9 (13.4)
Sex	Female	17 (25.4)
	Male	50 (74.6)
Years of Experience	≤ 5	53 (79.1)
	>5	14 (20.9)
Practice area	OPD pharmacy	22 (32.8)
	Emergency pharmacy	15 (22.4)
	Inpatient pharmacy	9 (13.4)
	ART pharmacy	15 (22.4)
	Store pharmacy	6 (9)

Key; OPD, Outpatient department; ART, Antiretroviral therapy

The four multi-item variables had good internal reliability (Cronbach’s alpha (α) ≥ 0.7) for both physicians and pharmacists, except role specification and relationship initiation among the pharmacists as indicated in Table 3.

**Table 3:** Reliability coefficients for PPCI domains and collaborative care of physicians and pharmacists working in the governmental hospitals of Jigjiga town, SNRS, Ethiopia, November 2020

	Cronbach’s alpha (reliability coefficient) )	
	**Physicians**	**Pharmacists**
Trustworthiness	0.721	0.711
Role specification	0.748	0.633
Relationship initiation	0.698	0.669
Collaborative care	0.751	0.697

Effective inter-professional communication among health care professionals needed for quality health care delivery and it would depend on the way they approach each other [31, 32]. The physicians responded that their communication with the pharmacist was two-way (25, 48.1%) and characterized by open communication (32, 61.5%). There was no significant difference between the mean scores of physicians and pharmacists on trustworthiness and role specification domains as shown in Table 4. The mean scores of trustworthiness and role specification in both professionals were comparable. However, pharmacists had a higher score in the relationship initiation and collaborative care domain. The result indicated that pharmacists had a greater likelihood to initiate relationships than physicians (p-value ≤ 0.05). Relationship initiation indicates the action of physicians or pharmacists to determine the needs of one another thereby facilitating relationship development.

**Table 4:** The mean score of PPCI domains and collaborative care of physicians and pharmacists working in the governmental hospitals of Jigjiga town, SNRS, Ethiopia, November 2020

Domains	Physician Mean (± SD)	Pharmacist Mean (± SD)	p-value
Trustworthiness	28.1 ± 4.7	28.3 ± 4.7	0.8
Role specification	22.85 ± 4.3	22.63 ± 4.8	0.8
Relationship initiation	8.10 ± 2.5	10.80 ± 4.0	0.0
Collaborative care	9.17 ± 3.9	10.66 ± 4.6	0.1

According to their responses, pharmacists reported a higher mean of collaboration (10.7 ± 4.8) than physicians (9.2 ± 3.9). However, both means showed that both professionals had less likelihood to collaborate according to the 7-point likert scale.

The multiple regression analysis showed that area of practice was significantly associated with collaboration, with the highest collaboration in internal medicine for the physicians and inpatient and ART pharmacy for the pharmacists as shown in Tables 5 and 6. This study also revealed that a significant (p < 0.05) association between collaborative care and the two PPCI domains (trustworthiness and relationship initiation for the physicians; role specification and relationship initiation for pharmacists).

**Table 5:** Multiple regression analysis of factors influencing the collaborative care for the pharmacists working in the governmental hospitals of Jigjiga town, SNRS, Ethiopia, November 2020

Independent variables	Beta coefficients	p-value
**Sex**	0.55	0.53
**Age category**		
18-25^a^	-0.67	0.67
26-35^a^	0.73	0.66
36-45^a^	-2.76	0.21
**Years of experience**		
≤ 5	0.21	0.88
**Area of practice**		
OPD pharmacy ^b^	-0.59	0.67
Emergency pharmacy ^b^	0.78	0.62
Inpatient pharmacy ^b^	3.84	0.03*
ART pharmacy ^b^	3.13	0.04*
Trustworthiness	-0.12	0.29
Role specification	0.32	0.01*
Relationship initiation	0.53	0.00*

^a^Age ≥ 46 is the reference group for the age classification; ^b^Drug store is the reference for practicing area of pharmacists; OPD, Outpatient department; ART, Antiretroviral therapy; *p < 0.05

**Table 6:** Multiple regression analysis of factors influencing the collaborative care for the physicians working in the governmental hospitals of Jigjiga town, SNRS, Ethiopia, November 2020

Independent variables	Beta coefficients	p-value
**Sex**	-1.63	0.08
**Age category in years**		
18-25^a^	-1.14	0.53
26-35^a^	-1.80	0.30
36-45^a^	-4.14	0.04*
Years of experience in years (≤ 5)^b^	-0.39	0.55
**Area of practice**		
Internal medicine ward^c^	3.66	0.01*
Pediatric ward^c^	0.35	0.67
Surgical ward^c^	0.18	0.83
OPD ward^c^	0.50	0.55
Emergency Ward^c^	-1.24	0.34
Trustworthiness	0.17	0.03*
Role specification	-0.05	0.50
Relationship initiation	0.62	0.01*

^a^Age ≥ 46 is the reference group for the age classification; ^b^Drug store is the reference for practicing area of pharmacists; OPD, Outpatient department; ART, Antiretroviral therapy; *p < 0.05

## Discussion

Evidence showed that collaboration between the physicians and pharmacists improves patient treatment outcomes [1, 33–36]. A recent systematic review showed that several studies investigating physicians-pharmacists collaboration using the CWR model and PPCI have focused on collaboration in community settings [37]. This study reported the collaboration between physicians and pharmacists of the two governmental hospitals of Jigjiga town, SNRS, Ethiopia.

In the current study, the physicians and the pharmacists had comparable mean scores for trustworthiness and role specification. However, pharmacists had 2.7 higher mean scores of relationship initiation than the physicians. In contrast, a study in Ethiopian teaching hospital [6] indicated that physicians had a higher score in all the domains of PPCI than the pharmacists. Other studies [5, 38] also determined higher mean score among physicians than pharmacists in all the domains of PPCI. The difference may be attributed to infancy of clinical pharmacy service in Ethiopia. Ethiopia has been known for its long track record of product-oriented pharmacy practice. The newly graduated clinical pharmacists always seek to start the clinical pharmacy service in their institution. But, physicians and other healthcare professionals were not well understood the role and responsibilities of pharmacists in patient care. The Ethiopian pharmacy curriculum produce competent graduate who can undertake innovative research, and disseminate demand-driven knowledge and pharmaceutical practice to the community, but the healthcare policy of the country offer them un proportional work area [39].

There have been various research studies in which collaborative working relationships between physicians and pharmacists and their benefits for patient care and disease outcome [16, 40, 41]. However, the current study indicated lower collaborative care between physicians and pharmacists and both encounter resistant colleagues to collaboration. Ethiopian physicians expect pharmacists to take initiative to introduce themselves and offering their collaboration in medication selection, drug regimen review and assess and manage drug’s adverse reaction during bedside rounds and morning classes. However, most physicians usually have short regular visits to their hospital pharmacy and drug information centers to get updated with newly arrived medications and drug information services that could build physician-pharmacist collaboration.

This study revealed that area of practice was significantly associated with collaboration, with the highest collaboration in internal medicine for the physicians and inpatient and ART pharmacy for the pharmacists. The internal medicine ward was the most common practice site where pharmacists and physicians have more contact, as clinical pharmacy service was initiated there. Studies indicated that collaboration can be achieved when the professionals have participated in common community organizations which allow more contact among them [30]. A similar result was reported from teaching hospitals of Ethiopia [6] and Iraq [5], but the highest collaboration in the pediatric ward.

The result of this study indicated that relationship initiation for both pharmacists and physicians’ had a significant association with the degree of collaboration. Similarly, a study in teaching hospitals of Ethiopia [6] indicated that relationship initiation was the factor influencing the degree of collaboration for the pharmacists. The study conducted in US [28] also showed that relationship initiation was the most influential factor supporting collaboration between pharmacists and physicians. Another study from Iraq [5] showed that physician’s relationship initiation had a significant positive relationship with collaboration.

A successful inter-professional relationship in health care practices based on formal organizational action [26]. Therefore, it is the professionals’ responsibility to initiate and maintain the relationship [39]. A successful collaboration could be created, primarily when physicians seek to establish professional relationships with pharmacists, given the greater power and influence of the physician in the healthcare settings. A relationship initiation also can be achieved by participation in a common community organization which is another way to allow contact between the professionals [30]. The latter may a probable reason for relationship initiation was higher in pharmacists than physicians. Pharmacists practicing in inpatient pharmacy are expected to participate in the morning discussion with physicians and a major round with the healthcare team to influence medication selection and improve patient outcomes. These create opportunities to contact and accelerate establishing collaboration.

The current study indicated that trustworthiness in the case of physicians and role specification in the case of pharmacists had a significant association with the degree of collaboration. Studies from USA [38] and Iraq [5] showed trustworthiness and role specification had a significant association with the degree of collaboration among the pharmacists. These studies [5, 38] also indicated that for both professionals, role specification had a significant positive association with the collaborative care. Trustworthiness has a vital role in the development of collaborative practice. When a pharmacist/physician shows his/her competence consistently, trust begins to build up [25]. A Study in the UK also found trustworthiness is vital to build rapport and foster the exchange between pharmacists and general practitioners [42]. Pharmacists must prove their drug expertise and competence through providing useful clinical recommendations that improve patient health to build trust and to support movement into collaboration. When mutual trust is built, the collaboration level will usually advance. Some physicians may not accept pharmacists’ intervention [30]. However, physicians’ responses indicated that building trust is a significant factor influencing the degree of collaboration.

Role specification also had a significant positive association with collaborative care. As professionals start working together, each may hold expectations about the other that is based on past experiences, stereotypes and educational background [43]. Roles for pharmacists include, taking a medication history, drug therapy management, developing a pharmaceutical care plan, providing recommendations to physicians, and patient follow-up and monitoring. When physicians and pharmacists jointly determine specific roles, the relationship is more likely to become collaborative. Pharmacists may encounter physicians who were resistant to collaboration and vice versa.

### Limitations of the study

The survey used a convenience sample, which might not represent the general population of hospital pharmacists and doctors. This study did not explore barriers to collaborative practice.

## Conclusions

The study showed that the collaboration between the physicians and pharmacists was sub-optimal. The result indicated that physicians-pharmacist collaboration was significantly influenced by the PPCI exchange variables. In the physicians’ point of view, trustworthiness and relationship initiation and in the pharmacist point of view, role specification and relationship initiation factors significantly influence the degree of physicians and pharmacists collaboration.

### Recommendation

Physicians and pharmacists need to exert more efforts to enhance this collaboration. Further qualitative study might be needed to search for factors affecting, barriers and how to develop collaborative practice to minimize drug-related problems and improve patient outcomes in Ethiopia.

## Supplementary Information

**Additional file 1.**

## Data Availability

All relevant data are within the paper. The SPSS data of individual participants are not permitted to be provided to other bodies, as indicated on ethical clearance. However, researchers who need further clarification can obtain anonymized data from the corresponding author on reasonable request.

## Abbreviations

ART: Antiretroviral therapy
CWR: Collaboration working relationship
GP: General practitioner
Gyn: gynecology
KH: Karamara Hospital
Obs: obstetrics
OPD: Outpatient department
PPCI: Pharmacist-physician collaboration index
SHYRH: Sheik Hassan Yebare Referral Hospital
