# Supplementary material for: Factors influencing the degree of physician-pharmacists collaboration within governmental hospitals of Jigjiga Town, Somali National Regional State, Ethiopia, 2020
Source: BMC Health Serv Res. 2021 Nov 24;21:1269. doi: 10.1186/s12913-021-07301-7 (PMC8611947; doi:10.1186/s12913-021-07301-7)
Supplement: Supplementary file 1 — Additional file 1. [file 12913_2021_7301_MOESM1_ESM.docx]

**Supplementary file 1**

**Physicians pharmacists collaboration instrument (PPCI) items**

**Trustworthiness**

1. The pharmacist is credible
2. Trust this pharmacist drug expertise
3. I can count on this pharmacist to do what he/she says
4. Communication between this pharmacist and myself is two way
5. I intend to keep working together with this pharmacist
6. My interaction with pharmacist is characterized by open communications of two parties

**Role specification**

1. This pharmacist and I negotiate to come to agreement on our activities in managing drug therapy
2. This pharmacist and I are mutually dependent on each other in caring for the patients
3. I will work with this pharmacists to overcome disagreement on his/her role in managing drug therapy
4. In providing patient care, I need this pharmacist as much as this pharmacists needs me
5. This pharmacist depends on me as much as I depend on him/her.

**Relationship initiation**

1. I spent time trying to learn how he/she can help you provide better care
2. Showed an interest in helping you to improve your practice
3. Provide information to you about specific patient

**Collaborative care measures**

1. I work with this pharmacists to plan the goals of drug therapy for our patients
2. Decision making responsibilities for our patients drug therapy are shared between this pharmacist and me
3. This is cooperation between this pharmacist and myself in managing the drug therapy of our patients
4. In making decision for our patients, physician-pharmacist opinions are considered
5. Decision making for our patients is coordinated between this pharmacist and me.
